# Reduction in the Motion Artifacts in Noncontact ECG Measurements Using a Novel Designed Electrode Structure

**DOI:** 10.3390/s23020956

**Published:** 2023-01-14

**Authors:** Jianwen Ding, Yue Tang, Ronghui Chang, Yu Li, Limin Zhang, Feng Yan

**Affiliations:** School of Electronic Science and Engineering, Nanjing University, Nanjing 210046, China

**Keywords:** noncontact ECG, ECG electrodes, motion artifact, adaptive noise reduction

## Abstract

A noncontact ECG is applicable to wearable bioelectricity acquisition because it can provide more comfort to the patient for long-term monitoring. However, the motion artifact is a significant source of noise in an ECG recording. Adaptive noise reduction is highly effective in suppressing motion artifact, usually through the use of external sensors, thus increasing the design complexity and cost. In this paper, a novel ECG electrode structure is designed to collect ECG data and reference data simultaneously. Combined with the adaptive filter, it effectively suppresses the motion artifact in the ECG acquisition. This method adds one more signal acquisition channel based on the single-channel ECG acquisition system to acquire the reference signal without introducing other sensors. Firstly, the design of the novel ECG electrode structure is introduced based on the principle of noise reduction. Secondly, a multichannel signal acquisition circuit system and ECG electrodes are implemented. Finally, experiments under normal walking conditions are carried out, and the performance is verified by the experiment results, which shows that the proposed design effectively suppresses motion artifacts and maintains the stability of the signal quality during the noncontact ECG acquisition. The signal-to-noise ratio of the ECG signal after noise reduction is 14 dB higher than that of the original ECG signal with the motion artifact.

## 1. Introduction

With the development of microprocessors and MEMS sensors, wearable daily ECG monitoring is widely used to detect occasional arrhythmias or to monitor cardiac medication or postoperative recovery. Wearable ECG data acquisition suffers from multiple noise interferences, including baseline drift, industrial frequency interference, inotropic interference, and motion artifact, etc. [1]. The noise interferences seriously affect the diagnosis of an ECG, especially the identification of the ST bands and QRS waveforms. Among them, motion artifact is the destructive interference, mainly from the relative motion and friction between the body and the electrode surface. The spectral range of motion artifact is very close to the ECG signal, which leads to the difficulty in suppressing the motion artifact.

To suppress motion interference, one approach is to optimize the wearable sensing hardware, including the electrodes and front ends. The principle of electrode optimization focuses on good contact between the skin, clothes, and the electrodes. Lin et al. discovered that the adoption of flexible electrodes could provide stable contact between the electrodes and the body both horizontally and vertically, which suppressed the motion artifact by reducing the relative motion between the electrodes and the body [2]. Investigations on the composition of flexible electrodes have been conducted to achieve better stability, thus improving the quality of the acquired data and reducing the motion interference [3,4,5,6]. Xu et al. designed a stretchable strain sensor based on a CNT/MXene/PDMS composite structure and AgNWs/GO hybrid transparent conductive electrodes, and both were used for acquiring an ECG signal at the wrist end [3,4]. Dong et al. designed a flexible MXene/CNTs/TPU composite film with a bilayer conductive structure through electrospinning and vacuum filtration manufacturing. The strain sensor was used for full-scale human motion detection, including substantial joint movements and subtle movements of the facial expression, respiration, and pulse beat, indicating the feasibility of the strain sensor in practical applications [5]. Ottenbacher et al. pointed out that the presence of common mode interference would make motion interference more pronounced by simulation [7]. Motion interference can also be suppressed through software procession, such as: optimized nonlinear filters [8], wavelet transform filtering [9], adaptive filtering [10], and blind signal separation [11]. Research on adaptive noise reduction has focused on two aspects; one is the selection of a reference signal that is coherent with the motion noise component in the signal source [12,13,14,15,16,17,18,19,20,21,22], and the other is the optimization of adaptive filtering algorithms [23,24,25]. In order to improve the noise reduction effect of the adaptive noise reduction, acquiring a reference signal from external sensors that are coherent with the motion artifact is a commonly used approach. There are many kinds of reference signals acquired by external sensors. In previous studies, the reference signals selected were mainly as follows: the acceleration signal, the electrode/skin impedance signal, the skin deformation signal, etc. Thakor et al. employed the pacemaker’s pulse signal as the reference signal, which was consistent with the ECG waveform, to suppress various noise interferences, including the motion artifact and industrial frequency interference [12]. Since not everyone wears a pacemaker in daily life, the employment of pacemaker pulses as a reference signal is not universally applicable. Luo et al. reduced the EMG interference and motion artifact by taking the potential difference between the ECG electrode and the adjacent point close to it as the reference signal [13]. Hamilton et al. adopted a strain sensor mounted on the electrode surface to measure the deformation of the electrode as the reference signal [14,15,16]. Ottenbacher et al. decreased the motion artifact by introducing the variation in the electrode/skin impedance as the reference signal [17,18,19,20]. Raya et al. measured the difference in the acceleration sensor values during exercise as the reference signal to reduce the motion artifact [19,20,21,22]. The adoption of deformation sensors, acceleration sensors, and impedance sensors all increase the hardware cost and the difficulty of the device design.

The aim of this paper is to propose a new method to suppress the noncontact ECG motion artifact, which adopts a newly designed bioelectric electrode structure for acquiring the ECG signal and the reference signal. The proposed method has significant advantages, such as a simple design, easy implementation, and low cost. The article is organized as follows. Firstly, based on the principle of noise reduction, the design of the electrode structure is introduced. Secondly, the multichannel signal acquisition system and electrodes are implemented, and the performance of the system is tested. Finally, experiments under normal walking conditions are carried out, and the suppression performance of the motion artifact is analyzed.

## 2. Design of Electrode Structure

The ECG signal is a nonstationary signal, and noise interference due to the patient’s EMG signal and respiration is mixed in with the detection of ECG signals; thus, motion artifacts also feature a high level of uncertainty. In order to eliminate the noise while preserving the minimum loss of the signal itself, adaptive filtering calls for a reference signal that is highly correlated with the noise [26].

**Figure 1:**
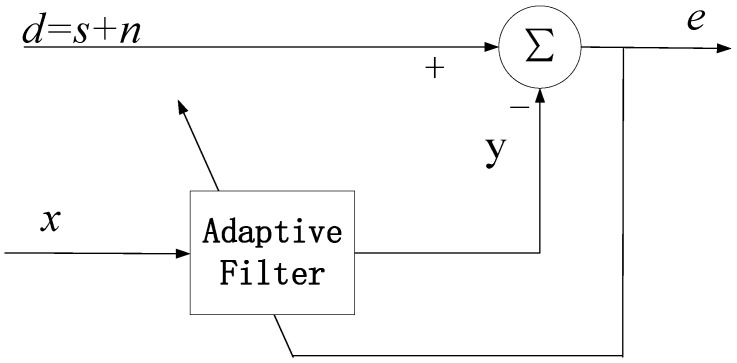
The adaptive noise canceling concept.

The block diagram of adaptive noise reduction is shown in Figure 1, where the input signal *d* contains the ECG signal *s* and the additive noise signal *n* [27,28]. The adaptive noise reduction method separates the noise signal from the main input signal *d* by introducing a reference signal *x*. It is assumed that the ECG signal *s* is uncorrelated with the reference signal *x* and the noise signal *n*, and the reference signal *x* and the noise signal *n* are correlated. The reference signal *x* is filtered to produce a filtered output *y*. Setting the order length of the filter as *L* and the weighting factor of the filter as *w*, the output y of the adaptive filter is expressed as

(1) $$y(k)=\sum_{l=1}^{L}w_{l}(k)x(k-l+1)$$

The difference between the main input signal *d* and the output *y* of the adaptive filter can be used as the error output *e*, which can be expressed by Equation (2).

(2) e=d−y=s+n−y

The mean square of *e* is as follows:

(3) $$\begin{array}{cc} E[e^{2}] & =E[{\left(s+n-y\right)}^{2}]\\ & =E[s^{2}]+E[{\left(n-y\right)}^{2}]+2E[s(n-y)] \end{array}$$

If the ECG signal *s* is not correlated with the reference signal *x*, Equation (3) can be simplified as:

(4) $$\begin{array}{cc} E[e^{2}] & =E[{\left(s+n-y\right)}^{2}]\\ & =E[s^{2}]+E[{\left(n-y\right)}^{2}] \end{array}$$

When *n − y* is infinitely close to 0, the error output *e* can be considered as the best least-squares estimate of the ECG signal *s*. The noise reduction performance is dependent on the level of correlation between the reference signal *x* and the noise signal *n*. Therefore, it is necessary to ensure that the reference signal *x* is highly correlated with the additive noise *n*, where the noise *n* will be suppressed by the adaptive filtered output *y*.

In order to obtain an appropriate reference signal *x*, a new electrode structure was designed. According to previous studies, the amplitude of the motion artifacts in the ECG signal is several times or even hundreds of times larger than the amplitude of the ECG signal. In noncontact ECG measurement systems, the amplitude of the ECG signal can be reduced while preserving the motion artifacts by increasing the insulation material thickness between the electrodes and the contact surface or by reducing the area of the electrodes, which provides a solution to obtain a reference signal barely containing an ECG signal.

**Figure 2:**
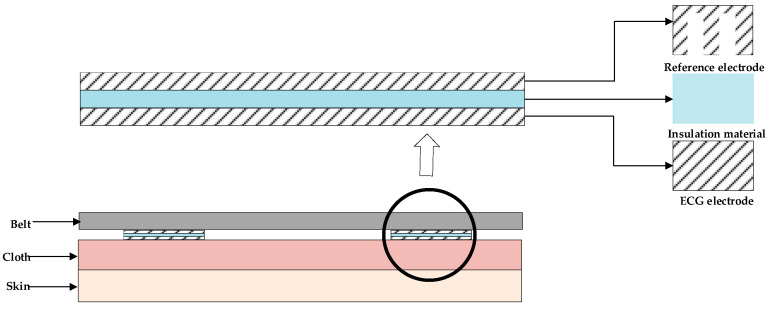
Schematic diagram of the wearable electrode structure.

Figure 2 shows the schematic diagram of the designed electrode structure. The carrier of the electrode is a bandage for a wearable chest ECG signal measurement. The reference signal acquisition electrode was isolated by insulating the material with the ECG signal acquisition electrode and designed with a comb-tooth shape to acquire motion artifacts, which reduced the electrode area and fit the contact surface as closely as possible. The ECG signal acquisition electrode was in the shape of a rectangle, facing the body and directly in contact with the cloth. The reference electrode, the ECG electrode, and the insulating material had the same outer contour size, thus improving the coherence of the reference signal and the motion artifacts in the ECG signal. The electrodes were all made of silver conductive fabric. The reference signal *x* was obtained from the difference between the two reference signal acquisition electrodes. The input signal *d* was obtained from the difference between the two ECG signal acquisition electrodes.

**Figure 3:**
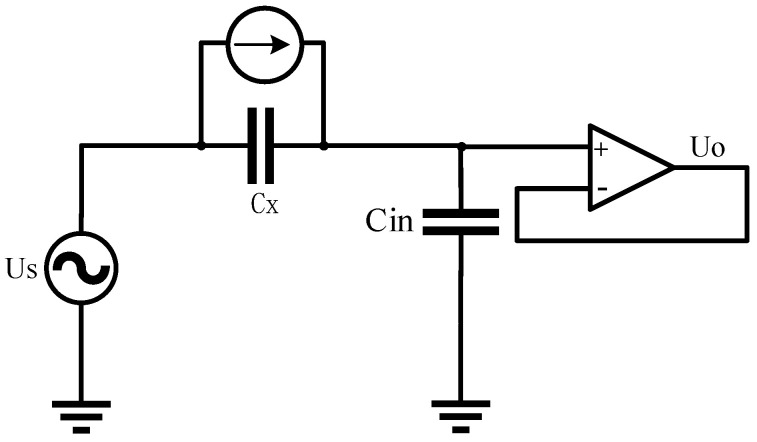
Simplified equivalent circuit diagram of the capacitive ECG measurements.

Figure 3 shows a simplified equivalent circuit diagram of the capacitive ECG measurements. The skin–electrode interface was modeled by a simple capacitor Cx, which can be considered a plate capacitor, with the electrode area *A* and the distance *D* between the electrode and the skin. Cin is the equivalent input capacitance of the acquisition front end. The voltage Us is a biosignal source, in this case the ECG. At the output of the amplifier, the voltage Uo can be measured, which is the reference input *x* from the reference electrodes. The motion artifacts are caused by the triboelectricity generated by friction and the variation in the skin–electrode interface. The friction between the skin, fabric, and electrode due to motion causes the charge accumulation on the textile electrode. Generally, the surface charge of the charged materials will decay with time. We adapted it here to include a current source *d*Q(t)/*d*t that represented the source of the motion artifact, where Q was the residual charge on the electrode.

For the reference electrodes, a comb-tooth shape can reduce the electrode area A, thus decreasing the value of capacitance Cx and reducing the ECG component of the reference signal *x*. On the other hand, the reference electrodes with a comb-tooth shape have the same outer contour size as the ECG electrodes and fit the contact surface as closely as possible. In this way, the charge variation *d*Q(t)/*d*t due to the friction between two kinds of electrodes is similar, and the reference signal *x* is highly correlated with the additive noise *n.*

## 3. Experimental Circuit System

### 3.1. Design of the Circuit System

**Figure 4:**
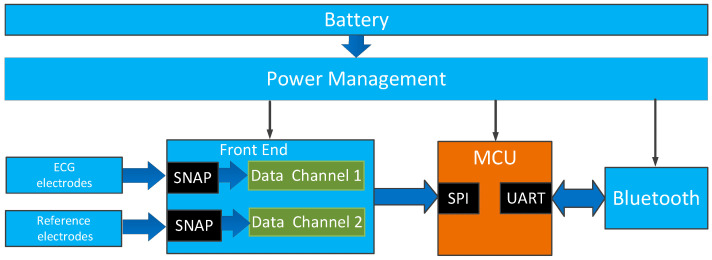
Block diagram of the system.

The diagram of the experimental circuit system is shown in Figure 4, including the ECG electrodes, reference electrodes, front end circuit, microcontroller, Bluetooth module, and the power management. Two pairs of stainless steel snap fasteners were used to connect the wearable electrodes with the front end. One pair of snap fasteners was used to connect the ECG electrodes, and the other was used to connect the reference electrodes. For the front end, ADS1298 was selected as the integrated ADC, where only two of the eight data channels were used. The front end was designed according to the proposed structure in reference [29]. Each channel was connected with an ultra-high input impedance sensor chip. The chip was characterized with an input capacitance of about 10 pF, and the input impedance was about 150 GΩ, which could efficiently acquire the noncontact ECG signal [29]. The microcontroller MSP430F5528 was used to sample the data from ADS1298. A 2.4 GHz Bluetooth module CC2540 was used to transfer data to the host computer, where the adaptive noise reduction processing and analysis of the acquired data were carried out. The power management circuit supplied the system using a rechargeable lithium polymer battery.

**Figure 5:**
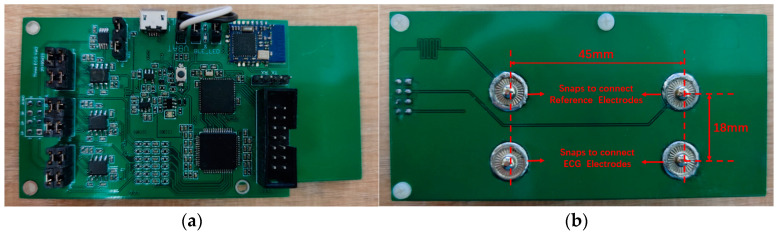
Printed system circuit board; (**a**) top layer of the circuit board; (**b**) bottom side of the circuit board.

The developed experimental circuit system is shown in Figure 5, with the size of 100 × 50 × 15 mm, where the components were placed on the top layer, and two pairs of snap buttons were placed on the bottom side. The diameter of the snap was 12 mm, and the distance between the two snaps for measuring the same type signal was 45 mm; the two sets of snaps were arranged in parallel with a spacing of 18 mm. The upper two snaps were for connecting the reference electrodes, and the lower two were for connecting the ECG electrodes. Each set of electrodes or snaps was symmetrically positioned on the left and right, and the symmetrical centerline was the vertical centerline of the human body. The snaps were used as the connectors between the electrodes and the printed board.

### 3.2. Performance of the Designed System

In order to obtain a high quality signal for identifying the ECG characteristics, the electrical characteristics of the system needed to meet certain specification requirements. In this section, we describe the testing of the electrical characteristics of the system, including the system noise, the common mode rejection ration (CMRR), and the frequency response. According to the International Standard IEC 60601-2-47 [30] and the American National Standard ANSI/AAMI EC13 [31], the requirements are shown in Table 1.

The CMRR measurement was carried out under the condition of the common input 50 Hz sinusoidal signal with an amplitude of 400 mV and the differential input 50 Hz sinusoidal signal with an amplitude of 4 mV. As shown in Table 2, the CMRR of the system achieved 100 dB, which is about 22 dB larger than the International Standard IEC 60601-2-47 requirement of 78 dB and 5 dB larger than the American National Standard ANSI/AAMI EC13 of 95 dB.

**Figure 6:**
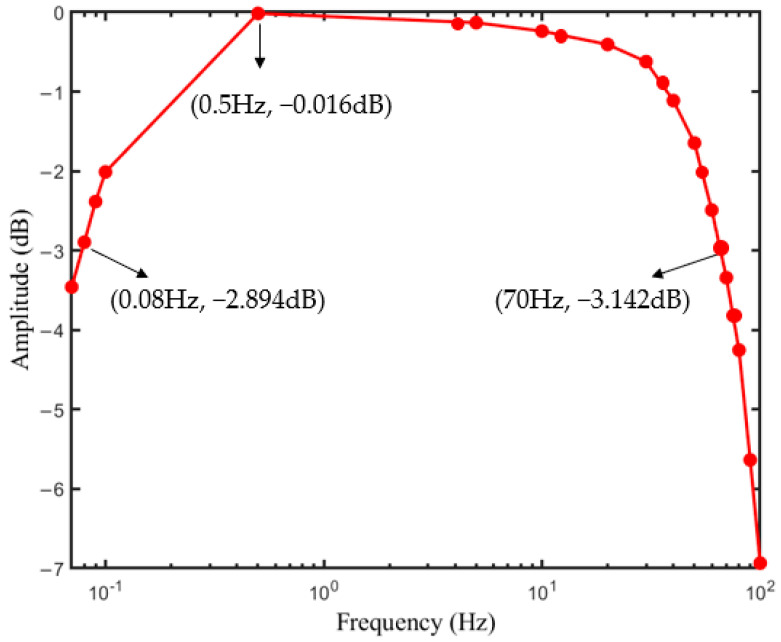
Frequency response measurement result.

The frequency response was obtained by introducing a 4 mV sinusoidal signal with a frequency from 0.07 Hz to 100 Hz. As shown in Figure 6, the bandwidth of the ECG system was from 0.08 Hz to 70 Hz, which met the standard frequency requirement 0.67–40 Hz of the ECG signal [32].

**Figure 7:**
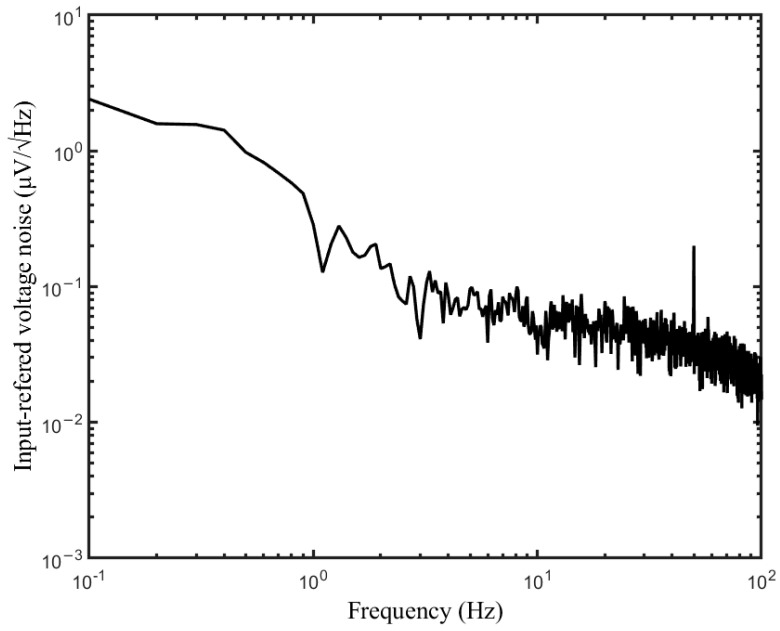
The input-referred voltage noise density.

The input referred voltage noise is shown in Figure 7, which was measured with the input terminals shorted to the ground. The input-referred voltage noise for the 0.1–70 Hz bandwidth was 1.32 μVRMS.

## 4. Experimental Results

**Figure 8:**
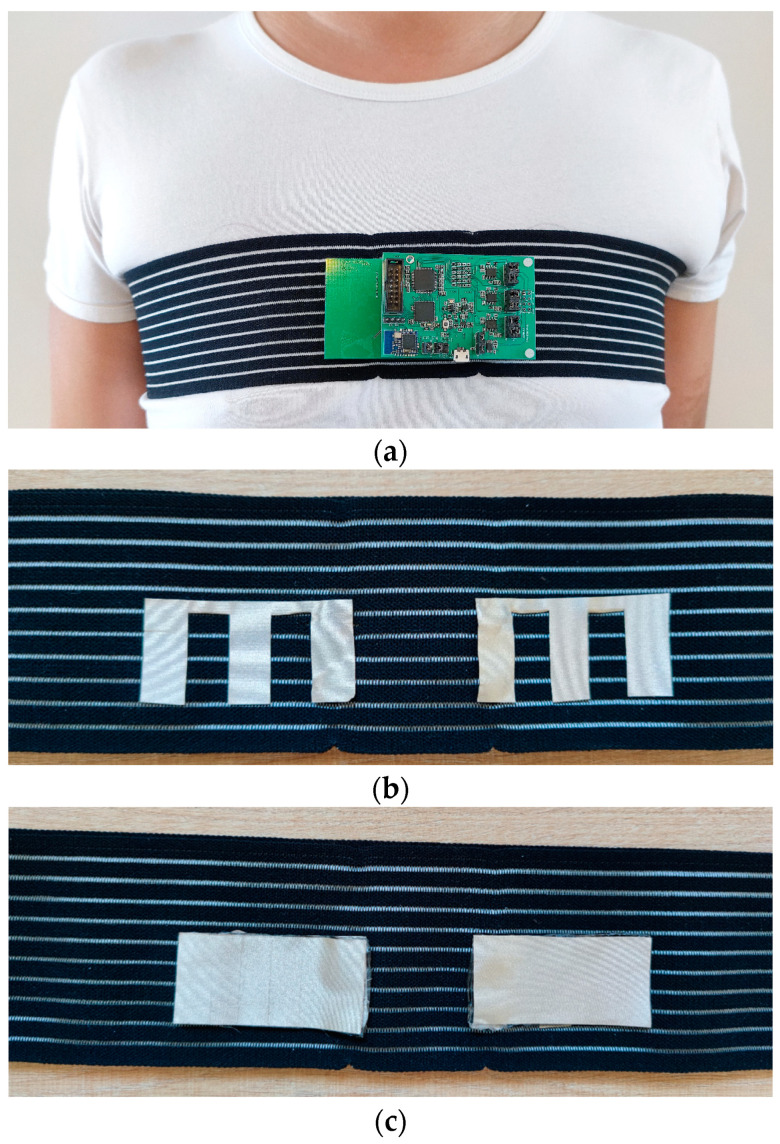
Measurement system; (**a**) tester wearing the experimental device; (**b**) reference electrodes; (**c**) ECG electrodes.

In this experiment, the experimental system was connected to a bandage with the snap fasteners, as shown in Figure 8a. Two sets of conductive silver fabric electrodes were stitched to the bandage. The reference signal acquisition electrodes were comb-tooth shaped, and the size of each comb tooth was 0.5 cm × 3 cm, as shown in Figure 8b. The ECG signal acquisition electrodes were rectangular electrodes with an electrode size of 6 cm × 3 cm, as shown in Figure 8c. The two types of electrodes were fixed and isolated from each other with 2-mm thick acrylic double-sided adhesive tape with the same size and shape as the ECG electrode. The reason for choosing acrylic double-sided adhesive tape was the softness of the acrylic material and its high level of adherence, which effectively reduced the relative motion between the ECG electrodes and the reference electrodes, as well as maintained the motion consistency, thus enhancing the coherence of the reference signal and motion artifacts. During the test, the bandage was tied to the volunteer’s chest, and the host computer was used to display and store the original ECG signal.

**Figure 9:**
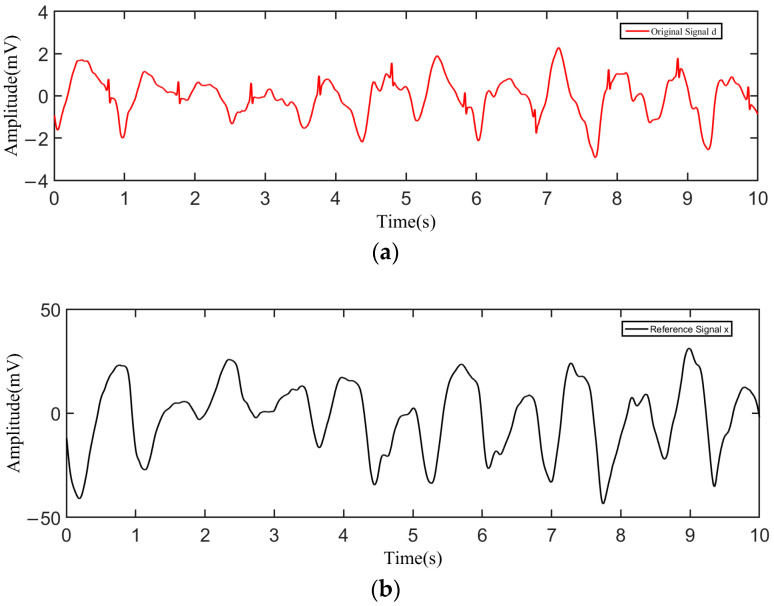
Original measurement results; (**a**) original signal; (**b**) reference signal.

**Figure 10:**
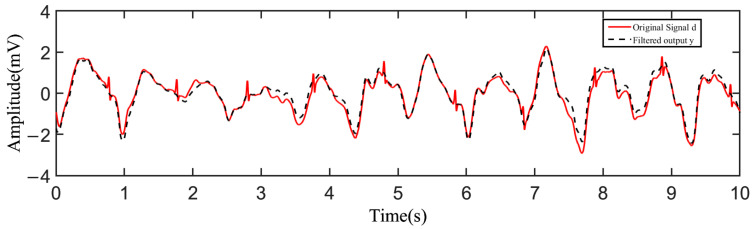
The comparison between the filtered output and the original signal.

**Figure 11:**
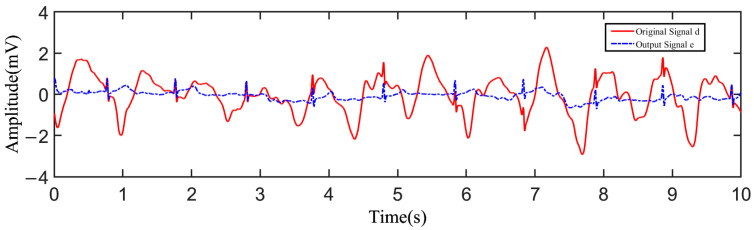
The contrast between the output signal and the original signal.

During the test, the tester wore a cotton T-shirt. After putting on the device, the tester performed the action of stepping in place with a speed of 1 m/s. The acquired raw data were transmitted to the host computer and plotted by Matlab as shown in Figure 9. Figure 9a shows the original signal *d* influenced by the noise obtained from one channel, and Figure 9b shows the reference signal *x* obtained from the other channel. As can be seen from the results, the original signal *d* was severely influenced by the motion artifacts, and the fluctuation in the baseline was remarkable. Through adaptive filtering, the filtered output *y* of the motion artifacts was derived. Figure 10 shows the comparison between the filtered output *y* and the original signal *d*, from which it can be found that the filtered output *y* fit the motion artifact smoothly. As shown in Figure 11, the output signal *e* was obtained by taking the difference between the original signal and the filtered output, where the motion artifact was significantly decreased, and the characteristics of the ECG waveform were more obvious.

For quantitative comparison, the signal to noise ratio (SNR) was calculated by Equation (5), where the (QRS) ECG_p-p_ is the peak-to-peak amplitude of R wave in a 120 ms span centered with the R wave, and the (T-P) noise_p-p_ is the peak-to-peak amplitude of the noise in a 40 ms T-P time interval span [33].

(5) $$\mathrm{SNR}=20\log\frac{(\mathrm{QRS}){\mathrm{ECG}}_{\mathrm{p-p}}}{{(\mathrm{T-P})\mathrm{noise}}_{\mathrm{p-p}}}$$

The SNR after adaptive noise reduction was 28.44 dB, which was about 14 dB larger than that of the original ECG signal, a value of 14.26 dB, which showed that the motion artifacts in the ECG signal were effectively suppressed by the proposed electrodes and the adaptive noise reduction processing method.

## 5. Conclusions and Discussion

A noncontact ECG offers good wearing comfort, which is well suited to be used for wearable long-time ECG monitoring. Compared to clinical ECG monitoring, a wearable ECG is more sensitive to motion artifacts. Adaptive noise reduction is an effective approach to suppress motion artifacts. In this paper, a novel ECG electrode structure was designed to acquire an ECG signal and reference signal simultaneously, avoiding the introduction of new sensors. The research employed a dual-channel data acquisition solution, which provided one more data acquisition channel than the conventional ECG measurements. By feeding data from both channels to an adaptive filter, the motion artifacts in the ECG acquisition were effectively suppressed.

Firstly, the design concept of the new ECG electrode structure and the principle of adaptive noise reduction were introduced. Secondly, a multichannel signal acquisition circuit system and wearable ECG electrodes were implemented, and the system performance was measured. Finally, experiments under normal walking conditions were conducted to verify the noise reduction effect. The ECG waveform characteristics of the obtained result after the noise reduction were obvious, and the motion artifacts were effectively suppressed. The signal-to-noise ratio of the noise-reduced ECG signal was 14 dB higher than that of the original ECG signal with the motion artifacts.

The proposed electrode design method was completely original and can be worn with commonly used bandages. The motion artifacts in the noncontact ECG were effectively suppressed during daily walking with the proposed design. In addition, since the solution did not involve additional sensors, the design was simple and can be applied to wearable applications, such as a chest strap, clothing, tag stickers, and other forms. By improving the SNR of the ECG signal, it could be beneficial for further waveform analysis, such as QRS identification, HRV analysis, etc. The research aimed to demonstrate the noise reduction effect of the electrode structure, so not much optimization was performed on the size of the electric board. In the future, we can optimize the PCB design to reduce the size of the electronic board and decrease the weight for better wearing comfort. Further we can also design a housing, so that the electronic board can be protected and the performance can be tested outdoors.

## Figures and Tables

**Table 1:** Performance Requirements.

Index	Test Conditions	Requirement
System noise	10 s of data	≤30 μV (p-v RTI)
CMRR	f = 50 Hz	≥78 dB (IEC 60601-2-47) ≥ 95 dB (ANSI/AAMI EC13)
Frequency response	−3 dB attenuation	0.67–40 Hz

**Table 2:** CMRR measurement results.

Mode	Input Signal		Output	Gain	CMRR (dB)
	Frequency (Hz)	Amplitude (mV)	Amplitude (mV)		
Differential	50	4	19.2	4.8	100
Common		400	0.0175	4.35 × 10^−5^	

## Data Availability

Not applicable.
